# Leukemia inhibitory factor inhibits erythropoietin-induced myelin gene expression in oligodendrocytes

**DOI:** 10.1186/s10020-018-0052-3

**Published:** 2018-09-27

**Authors:** Georgina Gyetvai, Cieron Roe, Lamia Heikal, Pietro Ghezzi, Manuela Mengozzi

**Affiliations:** Department of Clinical and Experimental Medicine, Brighton & Sussex Medical School, Brighton, BN1 9PS UK

**Keywords:** Central glia-4, Multiple sclerosis, Myelin oligodendrocyte glycoprotein, SOCS3, TLR2

## Abstract

**Background:**

The pro-myelinating effects of leukemia inhibitory factor (LIF) and other cytokines of the gp130 family, including oncostatin M (OSM) and ciliary neurotrophic factor (CNTF), have long been known, but controversial results have also been reported. We recently overexpressed erythropoietin receptor (EPOR) in rat central glia-4 (CG4) oligodendrocyte progenitor cells (OPCs) to study the mechanisms mediating the pro-myelinating effects of erythropoietin (EPO). In this study, we investigated the effect of co-treatment with EPO and LIF.

**Methods:**

Gene expression in undifferentiated and differentiating CG4 cells in response to EPO and LIF was analysed by DNA microarrays and by RT-qPCR. Experiments were performed in biological replicates of *N* ≥ 4. Functional annotation and biological term enrichment was performed using DAVID (Database for Annotation, Visualization and Integrated Discovery). The gene-gene interaction network was visualised using STRING (Search Tool for the Retrieval of Interacting Genes).

**Results:**

In CG4 cells treated with 10 ng/ml of EPO and 10 ng/ml of LIF, EPO-induced myelin oligodendrocyte glycoprotein (*MOG*) expression, measured at day 3 of differentiation, was inhibited ≥4-fold (*N* = 5, *P* < 0.001). Inhibition of EPO-induced *MOG* was also observed with OSM and CNTF. Analysis of the gene expression profile of CG4 differentiating cells treated for 20 h with EPO and LIF revealed LIF inhibition of EPO-induced genes involved in lipid transport and metabolism, previously identified as positive regulators of myelination in this system. In addition, among the genes induced by LIF, and not by differentiation or by EPO, the role of suppressor of cytokine signaling 3 (SOCS3) and toll like receptor 2 (TLR2) as negative regulators of myelination was further explored. LIF-induced *SOCS3* was associated with *MOG* inhibition; Pam3, an agonist of TLR2, inhibited EPO-induced *MOG* expression, suggesting that TLR2 is functional and its activation decreases myelination.

**Conclusions:**

Cytokines of the gp130 family may have negative effects on myelination, depending on the cytokine environment.

**Electronic supplementary material:**

The online version of this article (10.1186/s10020-018-0052-3) contains supplementary material, which is available to authorized users.

## Background

Oligodendrocytes (OLs), the myelinating cells of the central nervous system (CNS), produce the myelin sheath that provides physical protection and metabolic support to the axons and allows efficient conduction of action potential (Philips & Rothstein, 2017). In chronic inflammatory diseases, such as multiple sclerosis (MS), damage to OLs causes demyelination, impairs axonal function and leads to progressive degeneration of axons (Franklin & Gallo, 2014; Tauheed et al., 2016).

Remyelination, the process by which OL progenitor cells (OPCs) differentiate and mature to produce myelin that wraps demyelinated axons, can occur in the adult brain, where a wide-spread population of OPCs is present. Remyelination is usually highly efficient after injury and in the first stages of MS, but declines with aging and disease progression. Remyelination failure is a major determinant of progressive axonal degeneration and permanent neurological disability in chronic demyelinating diseases. Since OPCs are present in adult aging brain and in MS lesions, a block in differentiation and not a lack of OPCs seems responsible for remyelination failure (Franklin & Gallo, 2014; Kremer et al., 2015; Chamberlain et al., 2016).

The main immunomodulating drugs approved for MS can delay disease progression but do not prevent progressive disability since do not repair existing damage. Remyelinating therapies are needed. In the last years, several remyelinating strategies have been attempted, and drugs that inhibit negative signals (e.g. antibodies to LINGO-1) or provide positive stimulation (e.g. clemastine fumarate) are in the translational pipeline, but no remyelinating drugs are currently available (Kremer et al., 2015; Cadavid et al., 2017; Green et al., 2017; Bove & Green, 2017).

The observations that remyelination can be achieved in aging brain when appropriate exogenous factors are provided (Ruckh et al., 2012) and transplantation of neuronal precursors increases remyelination mainly by immunomodulatory mechanisms (Martino & Pluchino, 2006) suggest that direct administration of neuroprotective factors, as opposed to transplantation of stem cells, might be a good remyelinating strategy.

In the last 20 years, erythropoietin (EPO) has emerged as a potential candidate for neuroprotective and neuroregenerative treatment in injury and disease of the nervous system (Sargin et al., 2010). Interestingly, EPO improves cognitive performance in healthy animals and humans and in disease, including in MS (Ehrenreich et al., 2007; Robinson et al., 2018; Li et al., 2018). Although the mechanism is still largely unknown, we and others showed that EPO acts directly on OLs to increase myelination in vitro and in vivo (Sugawa et al., 2002; Cervellini et al., 2013; Hassouna et al., 2016; Gyetvai et al., 2017).

In a recent study aimed at identifying cytokines exhibiting protective and regenerative functions similar to EPO by “functional clustering”, leukemia inhibitory factor (LIF) emerged as one of the cytokines functionally similar to EPO (Mengozzi et al., 2014).

LIF is a member of the interleukin-6 (IL-6) cytokine family that signals through the LIF receptor (LIFR) and the cytokine receptor glycoprotein 130 (gp130), the latter shared with all the other cytokines of the IL-6 family, including ciliary neurotrophic factor (CNTF) and oncostatin M (OSM). LIF downstream signaling pathways include the JAK/STAT3, the PI3K/AKT and the MAPK/ERK pathways (Nicola & Babon, 2015; Davis & Pennypacker, 2018).

LIF is a pleiotropic cytokine that can have diverse and opposite effects on different cell types, resulting in stimulation or inhibition of cell proliferation, differentiation and inflammation (Nicola & Babon, 2015; Davis & Pennypacker, 2018; Slaets et al., 2010; Cao et al., 2011; Linker et al., 2008; Ulich et al., 1994). It is currently believed to play a crucial role in the response to injury, particularly in the CNS (Slaets et al., 2010). Its expression is increased in cerebral ischemia, spinal cord injury, Alzheimer’s disease, Parkinson’s disease, seizure and MS (Nicola & Babon, 2015; Slaets et al., 2010; Vanderlocht et al., 2006; Mashayekhi & Salehi, 2011).

In the CNS, LIF can act on immune, neuronal and glial cells (Davis & Pennypacker, 2018). Many studies point to a direct action on OLs. In particular, LIF is required in development for the correct maturation of OLs; in addition, in vivo and in vitro, both endogenous and exogenous LIF protect OLs from cell death and increase their proliferation, differentiation and maturation (Nicola & Babon, 2015; Davis & Pennypacker, 2018; Slaets et al., 2010; Stankoff et al., 2002; Ishibashi et al., 2006; Emery et al., 2006).

Studies in LIF knock-out mice and exogenous LIF administration have highlighted its protective action in many models of demyelination (Nicola & Babon, 2015; Davis & Pennypacker, 2018; Slaets et al., 2010; Emery et al., 2006; Marriott et al., 2008), suggesting the possible therapeutic use of LIF and LIF inducers in demyelinating diseases, including MS (Slaets et al., 2010; Vela et al., 2016; Metcalfe, 2018).

Coadministration of neuroprotective agents rather than a single agent may be more effective. In this regard, EPO was previously reported to synergise with insulin-like growth factor (IGF)-1 to protect against neuronal damage (Digicaylioglu et al., 2004; Kang et al., 2010).

We have previously used an in vitro model of myelination, CG4 OPC transduced to overexpress erythropoietin receptor (EPOR), to study the mechanisms by which EPO increases myelin gene expression (Gyetvai et al., 2017). Aim of this study was to investigate whether co-treatment with EPO and LIF was more effective than EPO alone and the mechanisms involved. Surprisingly, we found that LIF strongly inhibited EPO-induced myelination. By gene expression profiling, we investigated the mechanisms mediating LIF inhibitory effects at the early stage of the OL differentiation process.

## Methods

### Cell culture and generation of CG4 cells expressing EPOR

Rat CG4 OPC overexpressing the EPO receptor (CG4-EPOR) were generated and cultured as reported in our previous studies (Cervellini et al., 2013; Gyetvai et al., 2017). As previously shown, wild type CG4 do not express EPOR and do not respond to EPO (Cervellini et al., 2013). However, primary OLs express low levels of EPOR under physiological conditions (Sugawa et al., 2002), and EPOR is induced in the CNS in pathologies where EPO has protective functions (Siren et al., 2001); in particular, injury induces EPOR expression in OLs (Ott et al., 2015). By overexpressing EPOR in CG4 cells, we set up an in vitro system that allowed us to characterise the mechanisms mediating EPO differentiating and myelinating effects in OLs, mimicking an in vivo situation of injury or disease, where EPOR would be up-regulated.

CG4-EPOR cells, for simplicity referred to as CG4, were used throughout this study. Briefly, CG4 cells were cultured in poly-L-ornithine-coated 6-well plates (320,000 cells in 4 ml of medium per well). They were maintained at the progenitor stage by culture in growth medium (GM), consisting of Dulbecco’s modified Eagle medium (DMEM) (Sigma-Aldrich) supplemented with biotin (10 ng/ml), basic fibroblast growth factor (bFGF; 5 ng/ml), platelet-derived growth factor (PDGF; 1 ng/ml), N1 supplement (all from Sigma-Aldrich) and 30% B104-conditioned medium, obtained as previously reported (Cervellini et al., 2013; Gyetvai et al., 2017). After overnight culture, the cells were induced to differentiate into OLs by switching to differentiation-promoting medium (DM), consisting of DMEM-F12 (Invitrogen/ThermoFisher Scientific) supplemented with progesterone (3 ng/ml), putrescine (5 μg/ml), sodium selenite (4 ng/ml), insulin (12.5 μg/ml), transferrin (50 μg/ml), biotin (10 ng/ml), thyroxine (0.4 μg/ml) and glucose (3 g/l) (all from Sigma-Aldrich), as reported (Cervellini et al., 2013; Gyetvai et al., 2017). Undifferentiated cells are bipolar; after 2 days of differentiation the cells acquire about 90% of multipolar morphology. Differentiated CG4 cells express myelin proteins, including MOG, a marker of myelin deposition in these cells (Louis et al., 1992; Solly et al., 1996). After 3 h in DM, some of the cells were treated with recombinant human EPO (Creative Dynamics), recombinant mouse LIF (Sigma-Aldrich), recombinant rat OSM (Peprotech), recombinant rat CNTF (Peprotech), or Pam3CSK4 (Pam3; InvivoGen). Human EPO is approximately 80% homologous to rodent EPO, and it is biologically active on rat cells (Gyetvai et al., 2017). Mouse and rat LIF share 92% sequence homology (Willson et al., 1992), and mouse LIF is biologically active on rat cells (Takahashi et al., 1995).

### RNA extraction

For the microarray experiment, total RNA was extracted and analysed as reported, using the miRNeasy system and protocol (QIAGEN) (Gyetvai et al., 2017). For all the other experiments, total RNA was extracted with QIAzol (QIAGEN), following the instructions of the manufacturer, and RNA purity and concentration were determined using a NanoDrop ND-1000 (NanoDrop Technologies/ThermoFisher Scientific).

### RT-qPCR

Reverse transcription (RT) and real time qPCR were carried out as reported (Gyetvai et al., 2017; Mengozzi et al., 2012), using TaqMan® gene expression assays (Applied Biosystems/ThermoFisher Scientific) and Brilliant III qPCR master mix (Stratagene/Agilent Technologies). Gene expression was quantified using the ΔΔCt method, according to Applied Biosystems’ guidelines. Results were normalized to *HPRT1* expression (reference gene) and expressed as fold change (FC) or as log_2_ FC vs one of the control samples, chosen as the calibrator, as previously reported (Mengozzi et al., 2012).

### Microarrays

All experimental conditions were performed in quadruplicate; undifferentiated cells were cultured in quadruplicate but only 3 random samples were used for microarray analysis and all of the 4 samples for qPCR validation. Results from 27 arrays are analysed and presented in this study: 3 undifferentiated (undif) and 4 differentiated (dif), 4 differentiated+EPO (EPO), 4 differentiated+EPO + LIF (EPO + LIF) at each time point (at 4 h and 23 h of differentiation; 1 h and 20 h after treatment with EPO and LIF respectively). RNA was amplified, labelled and hybridised onto Single Colour SurePrint G3 Rat GE 8x60K Microarrays (AMADID:028279; Agilent) at Oxford Gene Technology, Oxford, UK. Following hybridisation, the arrays were scanned to derive the array images. Feature extraction software v10.7.3.1 was used to generate the array data from the images.

### Microarray data analysis

Raw data in standard format from the microarray experiment have been deposited in the Gene Expression Omnibus (GEO) database of the National Center for Biotechnology Information (NCBI) (Barrett et al., 2013) and are accessible through GEO Series accession number GSE84687 (http://www.ncbi.nlm.nih.gov/geo). Raw data were normalised and analysed using GeneSpring (Agilent) and Excel (Microsoft) softwares. Transcript expression levels (log_2_ of the gProcessed Signal) between the experimental groups were compared by Student’s *t* test, obtaining uncorrected *P* values. Subsequent multiple comparison corrections were performed using the Benjamini-Hochberg (BH) False Discovery Rate (FDR) procedure, obtaining adjusted *P* values (BH adj. *P* values). Fold change in the expression was calculated as the ratio between the average of the gProcessed Signals of the various groups and expressed as log_2_. Differences in expression with a BH adj. *P* value < 0.05 and an absolute fold change ≥1.5 (log_2_ fold change ≥0.58) were considered statistically significant.

Functional annotation and biological term enrichment was performed using the DAVID v6.8 database (Database for Annotation, Visualization and Integrated Discovery) available online (https://david.ncifcrf.gov) (Huang da et al., 2009). Categories with *P* values < 0.05 were considered significantly enriched.

Gene-gene interaction networks were visualised using the STRING v10.5 database (Search Tool for the Retrieval of Interacting Genes/Proteins) available online (http://string-db.org). STRING assigns to each reported functional association a confidence score, which is dependent on both the experimental method on which the functional association prediction is based, and on the reliability of computational approaches used for prediction. We used all active prediction methods, and a confidence score > 0.4.

## Results

### LIF induces *MOG* with a bell-shaped dose response curve

CG4 cells, a largely used in vitro model of myelination, can be differentiated to produce myelin proteins, including myelin basic protein (MBP), a marker of differentiation, and MOG, a marker of myelin deposition(Louis et al., 1992; Solly et al., 1996). In previous studies, we have validated this model and shown that expression of MOG mRNA correlated with production of the protein, measured by western blot (Cervellini et al., 2013). Therefore, in this study we measured MOG mRNA as a marker of myelination in differentiated CG4 cells.

**Fig. 1:**
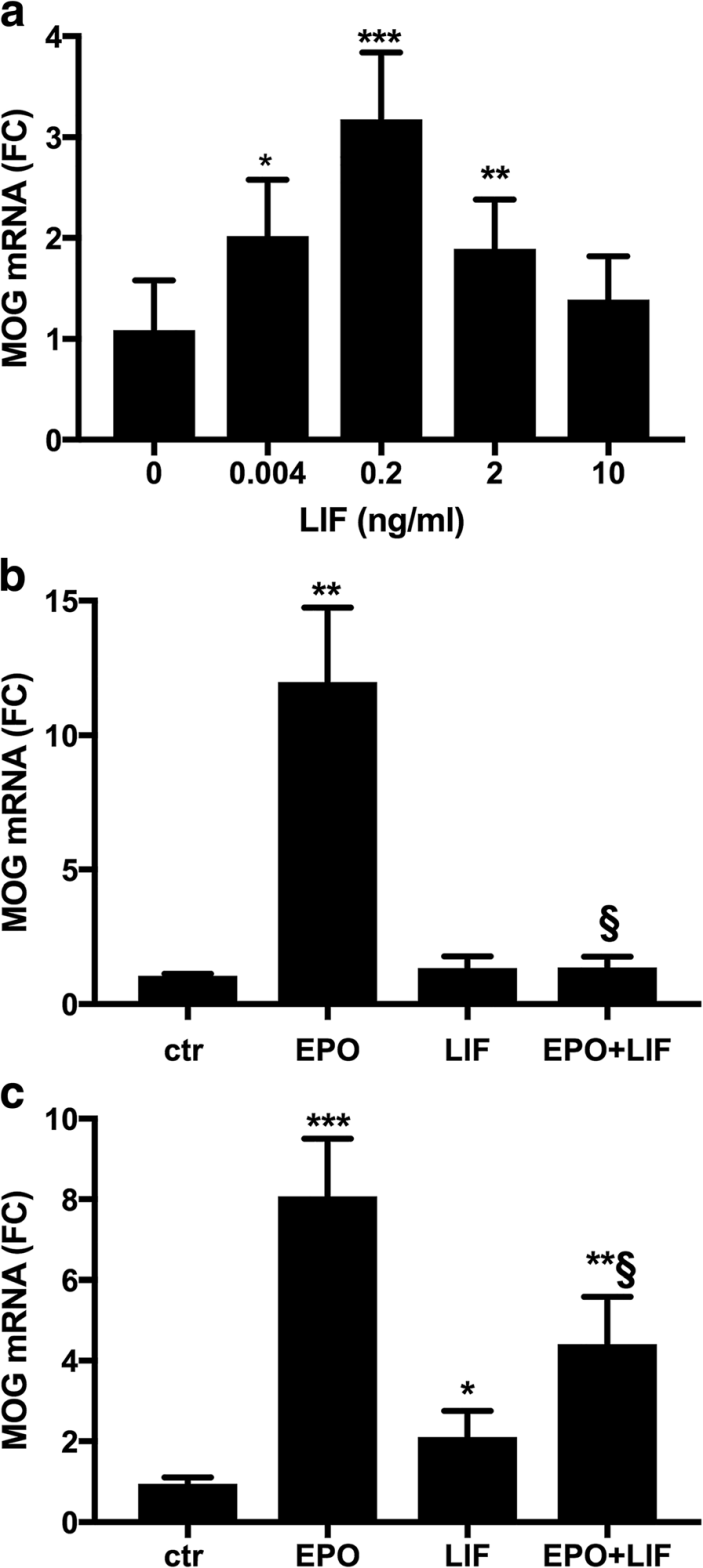
LIF induces MOG mRNA with a bell-shaped dose-response curve and inhibits EPO-induced MOG expression. Cells cultured for 1 day in growth medium (GM) were switched to differentiation medium (DM). After 3 h in DM the cells were treated with the indicated concentrations of LIF (**a**) or with or without EPO (10 ng/ml) and LIF (10 ng/ml, panel **b**; 0.2 ng/ml, **c**). MOG gene expression was measured by RT-qPCR at day 3 of differentiation. Results are expressed as fold change (FC) vs one of the control samples (no LIF in panel **a** and ctr in **b** and **c**). Data are the mean ± SD of seven samples from two independent experiments assayed in duplicate (**a**) or of quadruplicate samples assayed in duplicate and representative or five (**b**) or two (**c**) independent experiments; * *P* < 0.05, **P < 0.01, ***P < 0.001 vs control; § *P* < 0.01 vs EPO alone by two-tailed Student’s *t-*test

CG4 cells were differentiated for 3 days in DM with or without increasing concentrations of LIF ranging from 0.004 to 10 ng/ml. LIF increased *MOG* expression with a peak at 0.2 ng/ml and had no effect at the higher dose of 10 ng/ml, showing a bell-shaped dose response curve (Fig. 1a). In contrast, our previous results had shown that in these cells EPO still increased *MOG* expression at high doses, up to 400 ng/ml, although the expression plateaus after 10 ng/ml (Cervellini et al., 2013).

### LIF inhibits EPO-induced *MOG* expression

To investigate whether LIF synergised with EPO in increasing *MOG* expression, the cells were co-stimulated with EPO at 10 ng/ml and with LIF at 0.2 and 10 ng/ml. No synergistic or additive effect was observed; surprisingly, LIF markedly inhibited EPO-induced *MOG* expression at the high dose (10 ng/ml, Fig. 1b), and some inhibition was also observed at the low dose (0.2 ng/ml, Fig. 1c), which had a positive effect on *MOG* induction when added alone (Fig. 1a). Since EPO at high doses still increased MOG expression in these cells, as mentioned above and reported in a previous study (Cervellini et al., 2013), whereas LIF was less effective at high dose (10 ng/ml) than at low dose (0.2 ng/ml; Fig. 1a), these results suggest the LIF might induce a negative feedback that inhibits both its own and EPO’s pro-myelinating effects.

Of note, LIF at 10 ng/ml inhibited also EPO-induced myelin basic protein (MBP) expression at the same time point (at day 3 of differentiation): *MBP* mRNA as FC vs control, mean ± SD, *N* = 8; EPO: 3.7 ± 1.3, *P* < 0.001 vs control; EPO + LIF: 1.5 ± 0.4, *P* < 0.001 vs EPO alone by two-tailed Student’s *t*-test).

### LIF-induced changes in gene expression

To investigate the mechanisms by which LIF inhibits EPO-induced myelin gene expression, we performed a gene expression microarray study to identify the genes regulated by LIF in cells co-cultured with EPO and LIF, in which EPO-induced myelin gene expression was inhibited. We reasoned that co-culture with LIF might inhibit the effect of EPO by two mechanisms: i) inhibiting the expression of “positive regulators” of myelination increased by EPO; ii) increasing the expression of “negative regulators” of myelination, which are likely to be unchanged or decreased by differentiation or by EPO.

Analysis of the transcripts regulated by differentiation and further regulated by addition of EPO at 1 h and 20 h has been reported elsewhere (Gyetvai et al., 2017). Here we focussed on the genes regulated by LIF, selected by comparing EPO + LIF vs EPO at 1 h and 20 h and setting a fold change (FC) cut-off of 1.5 (log_2_ FC 0.58) and *P* value < 0.05 after applying the BH correction for multiple tests.

### Negative regulators of myelination induced by LIF at 1 h

**Fig. 2:**
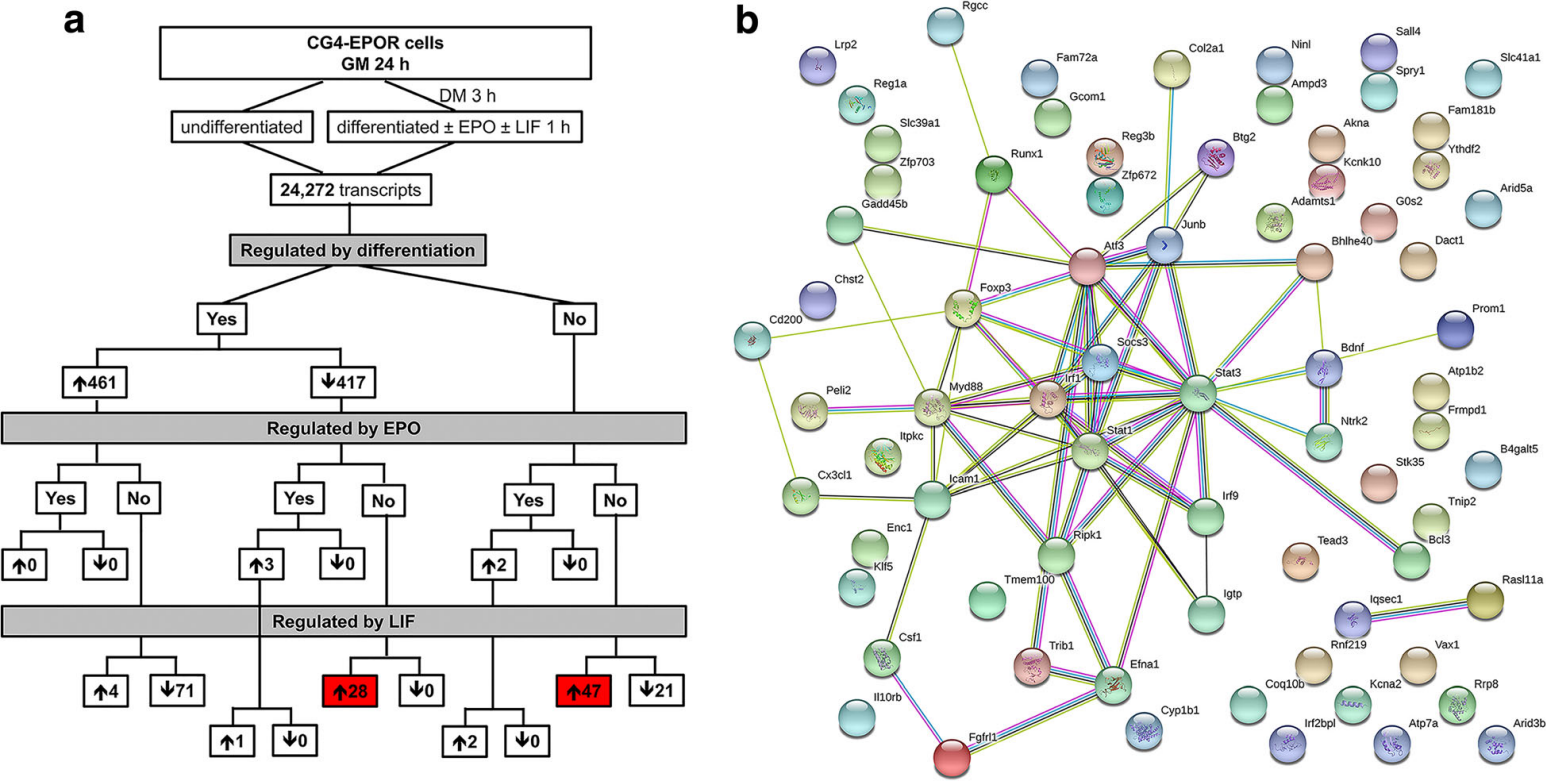
Genes regulated by LIF at 1 h. Cells cultured for 1 day in GM were switched to DM; after 3 h EPO with or without LIF was added and cells were incubated for further 1 h. **a** Flow chart. Genes regulated by differentiation were selected by comparing differentiating (4 h culture with DM) vs undifferentiated cells; genes regulated by EPO by comparing EPO-treated (1 h) vs untreated differentiating cells; genes regulated by LIF by comparing EPO + LIF-treated (1 h) vs EPO-treated cells. Cut-off for selection was FC of 1.5 (log_2_ FC of 0.58) and BH adj. *P* value < 0.05. The number of upregulated or downregulated genes resulting from filtering is indicated. Negative regulators induced by LIF and unchanged by differentiation or EPO (47) or decreased by differentiation (28) are highlighted in red. **b** Gene-gene interaction network of the putative negative regulators increased by LIF at 1 h. All the genes increased by LIF and either unchanged by differentiation or EPO (47 genes, Fig. 2a) or decreased by differentiation alone (28 genes, Fig. 2a) were analysed with the STRING software and the gene-gene interaction network was visualised. None of the genes increased by LIF were decreased by EPO at this time point. Different line colours represent types of evidence for association: green line, neighbourhood evidence; red line, fusion evidence; purple line, experimental evidence; light blue line, database evidence; black line, co-expression evidence; blue line, co-occurrence evidence; yellow line, text mining evidence. The full list of all the 75 genes and the relative expression changes induced by LIF and by differentiation are reported in Additional file 3

The gene expression profile of EPO-treated CG4 cells at 1 h and the effect of differentiation alone, previously reported (Gyetvai et al., 2017), is summarised in Fig. 2a; differentiation affected 878 genes, of which 461 were upregulated and 417 downregulated; treatment of differentiating cells with EPO for 1 h affected only 5 genes, which were all upregulated. Only 3 of these were affected and further increased by LIF (Fig. 2a and Additional file 1).

Since at the early time point LIF did not inhibit any EPO-induced gene, we focussed on the idea that it might induce negative regulators of myelination, whose expression would likely be either unchanged or decreased by culture in DM with or without EPO. When comparing EPO + LIF vs EPO, 82 genes were increased (Fig. 2a). Of these, 7 genes were excluded because they were also increased by differentiation alone (4, Additional file 2) or by EPO (3, Additional file 1). Therefore 75 genes that were either downregulated or not changed by differentiation, not altered by EPO and finally upregulated by LIF remained.

Network analysis of the remaining 75 genes (28 + 47, Fig. 2a) using the STRING database highlighted hubs centered on *STAT3* and *SOCS3* which included *Myd88*, part of toll-like receptor (TLR) signaling (Fig. 2b). A list of all the 75 genes, their fold change in expression levels by LIF (EPO + LIF vs EPO) and by differentiation (differentiated vs undifferentiated) is reported in Additional file 3.

### EPO-induced positive regulators of myelination inhibited by LIF at 20 h

**Fig. 3:**
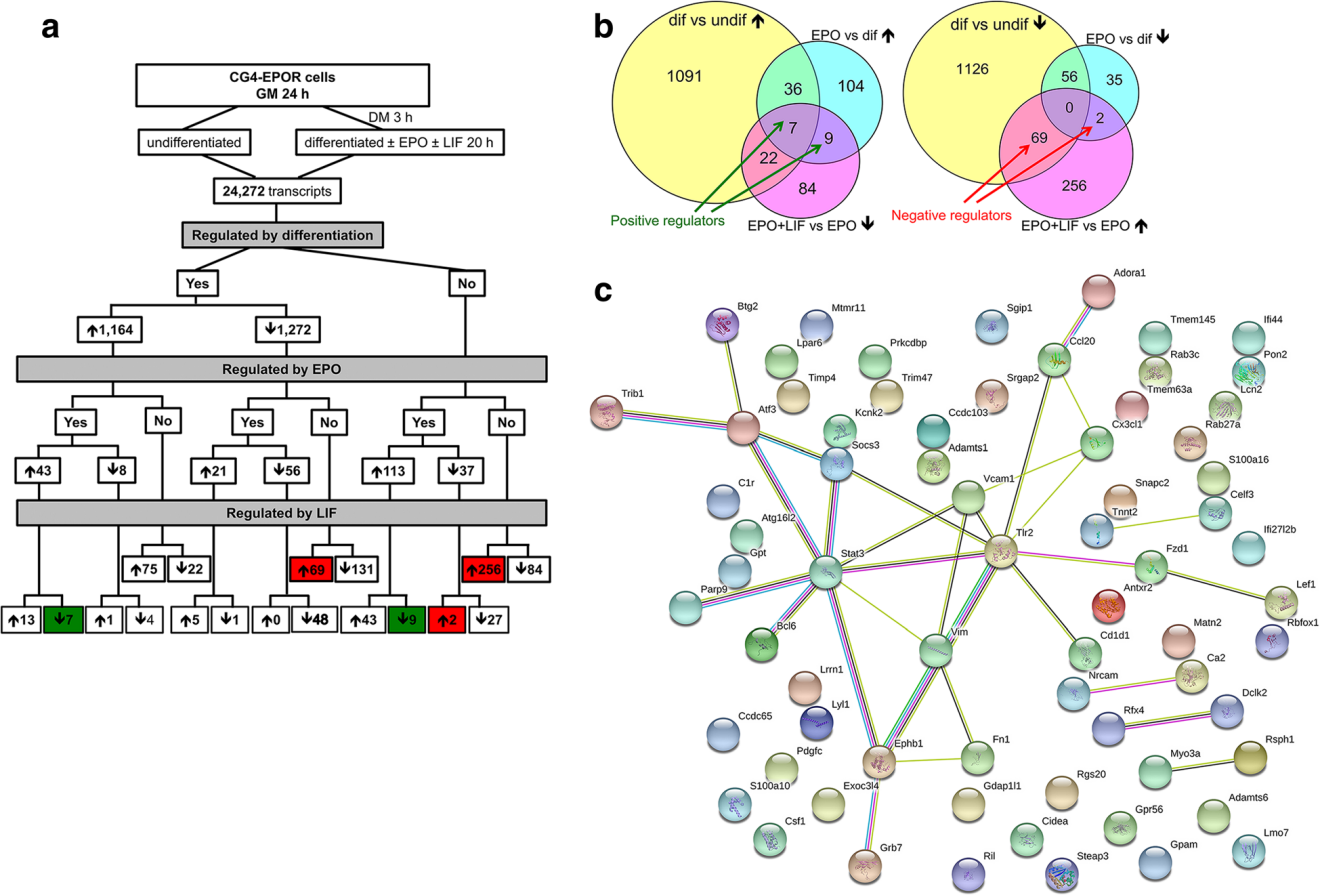
Genes regulated by LIF at 20 h. Cells cultured for 1 day in GM were switched to DM; after 3 h EPO with or without LIF was added for 20 h. **a** Flow chart. Genes regulated by differentiation, EPO and LIF were selected as in the legend to Fig. 2. Positive regulators induced by EPO and inhibited by LIF are highlighted in green (16, of which 7 induced also by differentiation). Negative regulators induced by LIF and unchanged by differentiation or EPO (256) or decreased by differentiation (69) or by EPO (2) are highlighted in red. **b** Venn diagrams showing positive regulators inhibited by LIF (left; EPO-induced genes unchanged or induced by differentiation, 9 and 7 respectively, green arrows) and negative regulators induced by LIF (right; 256 unchanged by differentiation or EPO; 69 and 2 decreased by differentiation or EPO respectively, red arrows). The genes changed in opposite directions by EPO and differentiation are not included in **b**. These are: 8 genes increased by differentiation but decreased by EPO and 21 genes increased by EPO but decreased by differentiation (**a**). In addition, the left diagram (positive regulators) does not include the genes decreased by LIF but also decreased by differentiation or EPO (4 + 1 + 48 + 131 + 27 = 211; **a**). The right diagram (negative regulators) does not include the genes increased by LIF but also increased by EPO or differentiation (13 + 1 + 75 + 5 + 43 = 137; **a**). Dif, differentiated; undif, undifferentiated. **c** Gene-gene interaction network of the putative negative regulators increased by LIF at 20 h. All the genes increased by LIF and decreased by differentiation alone (69 genes, **a**) or by EPO alone (2 genes, **a**) were analysed with the STRING software as described in the legend to Fig. 2. The full list of all the 71 genes is reported in Additional file 4

The gene expression profile of EPO-treated CG4 cells and the effect of differentiation at 20 h have been previously reported (Gyetvai et al., 2017). In Fig. 3a, the genes affected by LIF have been included.

At this time point EPO increased the expression of a number of genes, potential positive regulators of myelination, including 43 genes upregulated also by differentiation alone and 113 unaffected by differentiation. Addition of LIF decreased 7 of the 43 genes increased by EPO and differentiation, and 9 of the 113 genes increased only by EPO, as summarized in the Venn diagram in Fig. 3b (left). We focussed on the 16 putative positive regulators of myelination inhibited by LIF (green arrows, Fig. 3b), listed in Table 1. Functional annotation analysis of this subset of genes using the DAVID software highlighted enriched gene ontology biological process (GO:BP) and KEGG pathways categories involved in fatty acid transport, storage and oxidation; genes belonging to these categories included *CD36*, *Pnlip*, *Plin2*, *Ppargc1a* (Table 2). Of note, LIF inhibited also *Ptpre*, a protein tyrosine phosphatase which, among other effects, inhibits MAPK/ERK activation and that we previously identified as one of the top EPO-induced genes (Gyetvai et al., 2017).

**Table 1:** Genes increased by EPO and inhibited by LIF at 20 h

ProbeName	Gene	EPO + LIF vs EPO		EPO vs differentiation	
		Log_2_FC	BH adj.*P*	Log_2_FC	BH adj.*P*
A44P792784	*Htr2c*	−1.98	6.0E-04	5.14	5.1E-05
A64P128810	*RGD1565355*	−1.79	7.2E-04	5.11	9.3E-05
A64P113795	*LOC100365047*	−1.58	1.2E-02	2.06	3.9E-03
A64P057188	***Shroom2*** ^a^	−1.52	5.5E-03	1.73	1.6E-02
A64P054808	*CD36* ^a^	−1.47	1.1E-03	6.98	1.5E-04
A44P305482	*Ppargc1a*	−1.43	3.9E-03	1.48	1.6E-02
A44P335776	*Chodl*	−1.42	6.3E-03	1.89	5.9E-03
A44P158758	*Calcr*	−1.40	1.3E-02	1.78	1.9E-02
A64P15946	***Pmp2*** ^a^	−1.16	1.4E-03	5.24	1.5E-05
A64P025432	***LOC498829***	−1.04	6.0E-03	1.06	1.1E-02
A44P194803	***Baalc***	−1.03	3.1E-03	1.93	7.0E-04
A64P137130	*Ptpre*	−0.94	1.4E-02	4.01	3.5E-04
A44P254984	*Pnlip*	−0.89	4.8E-03	0.92	5.2E-03
A42P839964	*Plin2*	−0.79	8.7E-03	1.33	2.8E-03
A42P826938	***LRRTM1***	−0.63	3.9E-03	1.11	6.4E-04
A42P646991	***Mag***	−0.59	1.7E-02	1.34	7.9E-03

All the genes increased by EPO and inhibited by LIF at 20 h are listed. In bold the genes also increased by differentiation. The full list of the genes increased by EPO and differentiation at 20 h was previously reported (Gyetvai et al., 2017). ^a^Genes represented by 2 probes consistently changed by EPO in the same direction, of which only the most significantly changed one is shown

**Table 2:** Enriched KEGG pathways and GO:BP categories among the genes increased by EPO and inhibited by LIF at 20 h

Category	Term	Fold enrichment	Gene symbols	*P* value
KEGG	Fat digestion and absorption	87.7	*Pnlip, CD36, RGD1565355*	3.4E-04
KEGG	Adipocytokine signaling pathway	44.5	*CD36, Ppargc1a, RGD1565355*	1.3E-03
GO:BP	Intestinal cholesterol absorption	730.6	*PnlipP, CD36*	2.5E-03
KEGG	Insulin resistance	30.3	*CD36, Ppargc1a, RGD1565355*	2.9E-03
GO:BP	Response to drug	11.1	*CD36, Plin2, Htr2c, PPARGC1A*	3.7E-03
KEGG	AMPK signaling pathway	26.3	*CD36, PPARGC1A, RGD1565355*	3.8E-03
GO:BP	Cell surface receptor signaling pathway	22.8	*Calcr, CD36, RGD1565355*	6.1E-03
GO:BP	Long-chain fatty acid transport	243.5	*CD36, Plin2*	7.5E-03
GO:BP	Fatty acid oxidation	182.7	*CD36, Ppargc1a*	1.0E-02
GO:BP	Lipid storage	108.2	*CD36, Plin2*	1.7E-02
GO:BP	Response to lipid	97.4	*Pnlip, CD36*	1.9E-02
GO:BP	Receptor internalization	69.6	*Calcr, CD36*	2.6E-02
KEGG	Malaria	37.7	*CD36, RGD1565355*	4.5E-02

DAVID Functional Annotation Chart analysis showing the overrepresented GO:BP categories and KEGG pathways among the genes increased by EPO and decreased by LIF at 20 h. The fold enrichment and the significance of enrichment (*P* value) are reported

### Negative regulators of myelination induced by LIF at 20 h

As at the 1 h time point, we then searched for potential LIF-induced negative regulators at 20 h. These were selected by comparing EPO + LIF and EPO and setting a cut-off of FC > 1.5 (log_2_ FC > 0.58) and BH adj. *P* value < 0.05. As shown in Fig. 3a and in the Venn diagram in Fig. 3b (right), among the transcripts unchanged by either EPO and/or differentiation alone, we identified 256 genes increased by addition of LIF; out of 1272 genes decreased by differentiation, 69 genes were increased by LIF; among the 37 genes downregulated by EPO, 2 were increased by LIF. In total, 327 genes unchanged or decreased by differentiation or EPO were increased by LIF (full list Additional file 4).

STRING interaction analysis of the 71 genes induced by LIF and also decreased by differentiation (69) or EPO (2) (right red arrows, Fig. 3b), and therefore more likely to be putative negative regulators of myelination, highlighted a network of highly connected genes focused around *STAT3*, *SOCS3* and *TLR2* (Fig. 3c)*.*

### High expression of LIF-induced *SOCS3* is associated with reduced *MOG* expression

Since *SOCS3*, downstream of STAT3, was highly induced by LIF at both time points, and its expression in OLs can inhibit LIF-induced myelination in vivo in mice (Emery et al., 2006), we explored further its involvement in LIF-mediated inhibition of myelination.

The mRNA expression of *SOCS3* from the microarray experiment was validated by RT-qPCR using the same RNA used for the microarray experiment; inhibition of *SOCS3* by differentiation and induction by LIF at 1 h, reported in Additional file 3, were confirmed (*SOCS3* mRNA as log_2_ FC, mean ± SD, *N* = 4; dif vs undif: − 2.8 ± 0.2, *P* < 0.001; EPO + LIF vs EPO: 1.9 ± 0.3, *P* < 0.001 by two-tailed Student’s *t*-test).

**Fig. 4:**
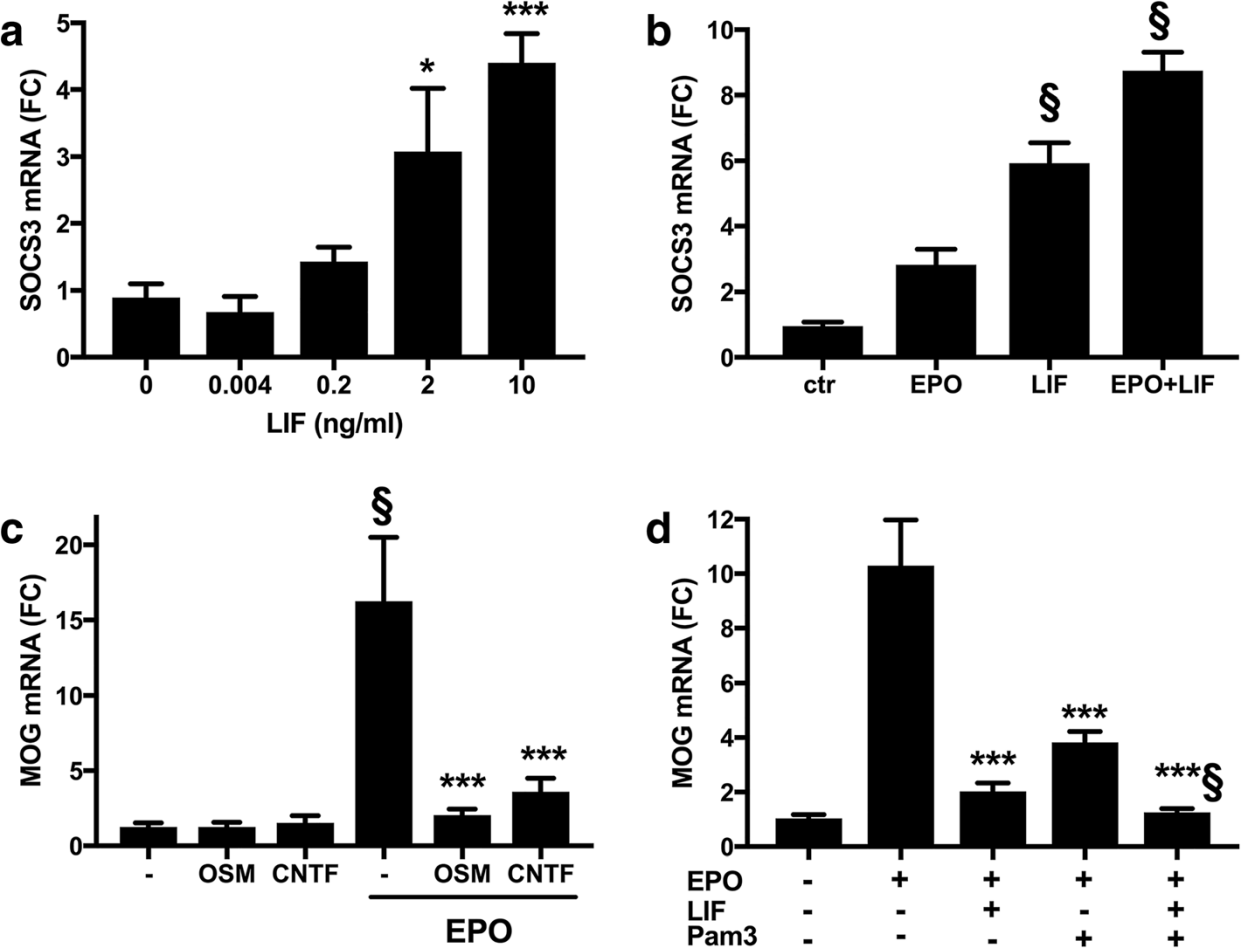
Role of SOCS3 and TLR2 in mediating LIF inhibition. **a-b** LIF induction of SOCS3 is associated with a reduction in MOG expression (shown in Fig. 1). Cells cultured for 1 day in GM were switched to DM; after 3 h they were treated with the indicated concentrations of LIF (**a**), or with or without EPO (10 ng/ml) and LIF (10 ng/ml; **b**). After 1 h, SOCS3 mRNA was measured by RT-qPCR. Results, expressed as fold change (FC) vs one of the control (ctr) samples (no LIF in **a**) are the mean ± SD of quadruplicate samples assayed in duplicate and are representative of two independent experiments; * *P* < 0.05, ***P < 0.001 vs control (no LIF); § *P* < 0.001 vs EPO by two-tailed Student’s *t-*test. **c** OSM and CNTF inhibit EPO-induced MOG expression**.** Cells cultured as above were treated with or without EPO (10 ng/ml) and OSM or CNTF (both at 6.5 ng/ml, equimolar concentrations to LIF 10 ng/ml). MOG gene expression was measured by RT-qPCR at day 3. Results, expressed as above, are the mean ± SD of eight samples from two independent experiments assayed in duplicate; *** *P* < 0.001 vs EPO alone; § *P* < 0.001 vs untreated by two-tailed Student’s *t-*test. **d** TLR2 engagement inhibits EPO-induced MOG expression. Cells were differentiated in the absence or in the presence of EPO (10 ng/ml), with or without LIF (10 ng/ml) or Pam3 (1 μg/ml), a TLR2/1 ligand. MOG expression was measured at day 3 by RT-qPCR. Results, expressed as above, are the mean ± SD of quadruplicate samples assayed in duplicate and are representative of two independent experiments; ***P < 0.001 vs EPO alone; § *P* < 0.01 vs EPO + LIF by two-tailed Student’s *t-*test

In independent experiments, *SOCS3* expression was dose-dependently induced by LIF (Fig. 4a). Furthermore, co-stimulation of EPO-treated cells with LIF which, as shown in Fig. 1b, inhibits EPO-induced *MOG* expression, induced high levels of *SOCS3* at 1 h (Fig. 4b).

The association between MOG inhibition and induction of high levels of SOCS3 was confirmed with OSM or CNTF, cytokines also belonging to the IL-6 family. At concentrations equimolar to the high dose of LIF (10 ng/ml), also OSM and CNTF inhibited EPO-induced MOG (Fig. 4c), and induced high levels of *SOCS3* at 1 h (*SOCS3* mRNA as FC vs control, mean ± SD, *N* = 4; OSM: 8.1 ± 1.7, *P* < 0.001; CNTF: 5.2 ± 1.7, *P* < 0.01 by two-tailed Student’s *t*-test). Of note, at a lower dose (0.13 ng/ml), equimolar to 0.2 ng/ml of LIF, OSM induced MOG expression, whereas CNTF had no effect (*SOCS3* mRNA as FC vs control, mean ± SD, *N* = 4; OSM: 3.2 ± 0.7, *P* < 0.001; CNTF: 1.4 ± 0.2, *P* = 0.19 by two-tailed Student’s *t*-test).

### TLR2 engagement inhibits EPO-induced MOG

Among the negative regulators induced by LIF, *TLR2* was also highlighted as a highly connected hub by STRING analysis at 20 h (Fig. 3c). Microarray expression of *TLR2* was validated by RT-qPCR using the same RNA used for the microarray experiment, confirming the inhibition of *TLR2* by differentiation and the very high induction by LIF at 20 h reported in Additional file 4 (*TLR2* mRNA as log_2_ FC, mean ± SD, *N* = 4; dif vs undif: − 1.5 ± 0.5, *P* < 0.01; EPO + LIF vs EPO: 3.6 ± 0.3, *P* < 0.001 by two-tailed Student’s *t*-test).

We therefore assessed the functional relevance of this finding using the TLR2 agonist Pam3. As shown in Fig. 4d, TLR2 activation inhibited EPO-induced *MOG* expression at the same extent as LIF and potentiated LIF inhibition.

## Discussion

Although there is ample evidence in the literature that LIF and other cytokines of the IL-6 family, including CNTF, have pro-myelinating activities in vivo and in vitro (Nicola & Babon, 2015; Davis & Pennypacker, 2018; Slaets et al., 2010; Metcalfe, 2018), we report here that LIF can inhibit myelination in vitro. Specifically, in CG4 OPC induced to differentiate into OLs in the presence of EPO, co-treatment with LIF inhibited EPO-induced *MOG* expression. Of note, LIF inhibition was observed in CG4 cells transduced to overexpress EPOR, and therefore optimised to respond to EPO. We had previously used this in vitro system to study the mechanisms by which EPO increased myelin gene expression (Gyetvai et al., 2017), using *MOG* as a readout since its expression is associated with myelin deposition in these cells (Solly et al., 1996). Compared to cells incubated in DM alone, treatment with EPO consistently induced high levels of *MOG* expression, which were strongly inhibited by LIF. The effect was more marked at high LIF concentrations (10 ng/ml), but inhibition was also noted at lower concentrations (0.2 ng/ml), which per se could slightly increase *MOG* expression. All together these observations highlight the strength of the inhibitory effect of LIF.

Our data may seem in contrast with many studies observing LIF pro-myelinating effects (Nicola & Babon, 2015; Davis & Pennypacker, 2018; Slaets et al., 2010; Metcalfe, 2018). However, no effect of LIF on OL differentiation had been previously described (Barres et al., 1993; Kahn & De Vellis, 1994; Park et al., 2001); interestingly, one study reported inhibitory effects of high LIF doses (more than 5 ng/ml) on OPC differentiation (Ishibashi et al., 2006). The ability of LIF to inhibit the pro-myelinating effects of other cytokines had not previously been reported.

LIF activates STAT3, which has a key role in myelination (Steelman et al., 2016). However, LIF signaling is tightly regulated. LIF-induced SOCS3, downstream of STAT3, inhibits STAT3 phosphorylation and excessive induction of inflammatory genes (Yasukawa et al., 2003), and is one of the main mechanisms through which LIF inhibits IL-6-induced differentiation of T helper (Th)17 cells (Cao et al., 2011). In the present study, LIF-induced *SOCS3* expression was associated with a reduction of EPO-induced *MOG* at high concentration of LIF. In addition, also OSM and CNTF, cytokines of the IL-6 family, used at equimolar LIF concentrations at which they induced similar levels of SOCS3 as compared to LIF (reported above in the Results section), inhibited EPO-induced MOG expression. These observations, together with previous results documenting increased myelination in SOCS3 knock-out mice (Emery et al., 2006), suggest that SOCS3 might play a role in LIF inhibition of *MOG* expression. SOCS3 induction might explain the lower levels of *MOG* observed at high doses of LIF compared to low dose, and inhibition of EPO-induced *MOG*. Of note, SOCS3 can inhibit EPO-induced STAT5 activation (Sasaki et al., 2000; Bachmann et al., 2011).

We investigated whether LIF might directly inhibit the expression of positive regulators of myelination induced by EPO. By gene expression profiling, we found that LIF downregulated genes involved in lipid transport and metabolism previously found to be increased by EPO, including *CD36*, *Ppargc1a*, *Pnlip* and *Plin2* (Gyetvai et al., 2017). Preferential downregulation of these genes by LIF strengthens the hypothesis that they might have a role in mediating EPO myelinating effects.

LIF inhibitory effects reported here cannot exclusively be correlated with an action on differentiated cells; LIF might also act on undifferentiated cells.

In this regard, LIF inhibited *PTPRE*, a tyrosine phosphatase induced by EPO that, among other effects, inhibits MAPK/ERK phosphorylation. We had previously shown that inhibitors of ERK in this system potentiate myelination, in support of the hypothesis that activation of ERK might sustain proliferation of OPCs and inhibit the start of differentiation (Gyetvai et al., 2017). Both EPO and LIF can induce ERK activation (Gyetvai et al., 2017; Nicola & Babon, 2015). However, EPO induces the feedback inhibitor *PTPRE*. Inhibition of *PTPRE* by LIF might prolong ERK activation in OPCs, inhibiting differentiation.

In addition, other than being pro-myelinating cytokines, LIF and other members of the IL-6 family, such as CNTF, are essential in development for inducing astrocyte differentiation. LIF can also increase astrocyte differentiation in vitro, although the presence of extracellular matrix factors may be required (Nicola & Babon, 2015). CG4 cells are bipotential OL type-2 astrocyte (O-2A) progenitors that can be induced to differentiate into type-2 astrocytes or into mature OLs (Louis et al., 1992; Solly et al., 1996). In primary OLs and CG4 cells LIF can induce the astrocyte marker GFAP (Kahn & De Vellis, 1994; Gresle et al., 2015), an observation that we have confirmed (Additional file 4). It is therefore possible that LIF, if present at the very early stages of the OL differentiation process, could interfere by inducing astrocyte differentiation. Although this is a very controversial issue, the presence of O-2A progenitors in vivo, and even in pathological conditions, has been suggested (Franklin & Blakemore, 1995; Virard et al., 2006).

Among the possible negative regulators induced by LIF, we noticed components of the TLR pathways, including TLR2 and Myd88, an adaptor protein used by almost all TLRs. Other than microbial products, the TLRs recognize endogenous danger-associated molecular patterns (DAMPs) released from injured tissues which regulate inflammatory responses (Lee et al., 2013). All cells of the CNS express the TLRs, including OLs which preferentially express TLR2 and TLR3 (Bsibsi et al., 2002; Sloane et al., 2010). TLR2 is upregulated in experimental models of MS and in MS demyelinating lesions, where it is also expressed by OLs (Sloane et al., 2010; Zekki et al., 2002; Esser et al., 2018); TLR2 activation inhibits OL maturation, an effect not shared by all TLRs (Sloane et al., 2010). We show here that TLR2 is functional in OLs, and its activation inhibits myelin gene expression.

Whether TLR2 has a role in mediating LIF inhibitory effects will of course depend on the presence of TLR2 ligands. TLR2, by forming homodimers and heterodimers with TLR1 or TLR6, can bind a broad range of ligands, including Gram-positive bacterial cell wall components, endogenous DAMPs such as heat shock proteins (HSPs) and high mobility group protein B1 (HMGB1), and fragments of extracellular matrix (ECM) molecules, such as hyaluronan (Piccinini & Midwood, 2010; Miranda-Hernandez & Baxter, 2013). Of note, TLR2 ligands, including hyaluronan, HMGB1 and peptidoglycan, a component of Gram-positive bacteria, have been detected in EAE and in MS lesions (Back et al., 2005; Visser et al., 2006; Andersson et al., 2008), suggesting that LIF-induction of TLR2 in OLs might actually lead to inhibition of remyelination.

Although LIF has an important role in promoting myelination (Slaets et al., 2010; Stankoff et al., 2002; Metcalfe, 2018), its pleiotropic nature, and its ability to induce proliferation inhibiting differentiation or vice versa, may result in negative myelinating effects at certain stages of the myelination process, likely when undifferentiated OL progenitors should stop proliferating and start differentiating. In pathological conditions, including MS, remyelination, especially at later disease stages, is insufficient to re-establish motor and cognitive performance. MS lesions may contain large numbers of poorly differentiated OPCs and immature OLs, suggesting that in many cases the main cause of remyelination failure is not a lack of OPCs, but rather an inability of these cells to differentiate into mature myelin producing cells (Franklin & Gallo, 2014; Kremer et al., 2015; Chamberlain et al., 2016). The presence of LIF in MS lesions (Vanderlocht et al., 2006) might contribute to inhibit OPC differentiation and remyelination.

Moreover, our findings show that, when considering the action of cytokines on myelination, one should consider that they act on a tightly regulated network, where each cytokine can affect the action of another. Identifying these regulatory networks may be important as different cytokines may be up- or down-regulated in disease conditions and this may have pharmacological relevance when cytokines are administered as neuroprotective or neuroreparative agents. Although the effectiveness of EPO in MS is unclear and recent clinical trials have not shown an efficacy (Schreiber et al., 2017), research is still active on EPO mimetics or derivatives with different biological properties (Culver et al., 2017; Bonnas et al., 2017); clinical trials with EPO in optic neuritis are ongoing after positive indications from phase 2 trials (Suhs et al., 2012; Diem et al., 2016) and its use to improve traumatic brain injury is still open (Counter et al., 1994). Likewise, there is interest in the potential use of LIF in the therapy of MS (Slaets et al., 2010; Metcalfe, 2018). The tight regulation of LIF signaling pathways that might negatively affect remyelination, shown here, needs to be taken into account in designing combination therapies and dose-finding studies. Additionally, increased blood and cerebrospinal fluid levels of LIF (Mashayekhi & Salehi, 2011), IL-11 (Zhang et al., 2015), CNTF (Massaro et al., 1997) and IL-6 (Wullschleger et al., 2013) have been found in MS patients, thus raising the possibility of them affecting the response to EPO.

Of course we should bear in mind the limitations of our study. The use of a cell line, although largely used for basic studies on myelination, limits the external validity of our findings, and only in vivo experiments in models of demyelination could indicate the in vivo relevance of the pathways that we have identified.

## Conclusion

This study reports that the IL-6 family cytokine LIF can inhibit EPO-induced myelin gene expression in OLs. LIF’s promyelinating effects have long been known, but controversial results have also been reported. The pleiotropic activities of LIF, which can inhibit or stimulate proliferation or differentiation and exhibit inflammatory or anti-inflammatory action, together with the tight inhibitory feedback mechanisms that regulate its signaling pathways, and its ability to induce negative regulators, such as TLR2, can translate into inhibition of myelination, depending on the stage of OL differentiation and on the cytokine environment. Further studies on the mechanisms by which endogenous cytokines positively and negatively affect myelination may lead to the identification of therapeutic targets and new drugs essential to improve remyelination in demyelinating diseases.

## Additional files

Additional file 1: Genes increased by EPO in differentiating cells at 1 h. Genes changed more than 1.5-fold (absolute log_2_ FC > 0.58), BH adj. *P* value < 0.05 in EPO-treated vs untreated differentiating cells are listed; ns = not significant. There were no genes decreased by EPO at this time point. The relative change in differentiating (dif) vs undifferentiated (undif) cells and in EPO + LIF vs EPO-treated cells are also reported. *Represented by 2 probes consistently increased by EPO of which only the most significantly changed one is shown (xlsx file). (XLSX 10 kb)

Additional file 2: Genes increased by LIF and by differentiation at 1 h. These genes have been identified by comparing EPO + LIF vs EPO and differentiating (dif) vs undifferentiated (undif) cells, setting a threshold of log_2_ FC ≥ 0.58 and BH adj. *P* value < 0.05. *Represented by 2 probes consistently increased by LIF of which only the most significantly changed one is shown (xlsx file). (XLSX 10 kb)

Additional file 3: Genes increased by LIF and unchanged or decreased by differentiation or EPO at 1 h. The genes increased more than 1.5-fold (log_2_ FC ≥ 0.58), BH adj. *P* value < 0.05 in EPO + LIF vs EPO-treated differentiating cells are listed; ns = not significant. For genes represented by 2 probes (*) consistently increased by LIF, only the one increased more significantly is shown (xlsx file). (XLSX 20 kb)

Additional file 4: Genes increased by LIF and unchanged or decreased by differentiation or EPO at 20 h. The genes increased more than 1.5-fold (log_2_ FC ≥ 0.58), BH adj. *P* value < 0.05 in EPO + LIF vs EPO-treated differentiating cells are listed; ns = not significant. For genes represented by 2 probes (*) consistently increased by LIF, only the one increased more significantly is shown (xlsx file). (XLSX 63 kb)

## Abbreviations

BH: Benjamini-Hochberg
CD36: Cluster of differentiation 36
CG4: Central glia-4
CNS: Central nervous system
CNTF: Ciliary neurotrophic factor
DAMP: Damage-associated molecular patterns
DAVID: Database for Annotation, Visualization and Integrated Discovery
DM: Differentiation medium
EPO: Erythropoietin
EPOR: Erythropoietin receptor
ERK: Extracellular signal-regulated kinases
GM: Growth medium
GO:BP: Gene ontology biological process
gp130: Glycoprotein 130
HPRT1: Hypoxanthine phosphoribosyltransferase 1
HSP: Heat shock protein
JAK: Janus kinase
LIF: Leukemia inhibitory factor
LIFR: Leukemia inhibitory factor receptor
MAPK: Mitogen-activated protein kinase
MOG: Myelin oligodendrocyte glycoprotein
MS: Multiple sclerosis
Myd88: Myeloid differentiation primary response 88
O-2A: Oligodendrocyte-type-2 astrocyte
OL: Oligodendrocyte
OPC: Oligodendrocyte progenitor cell
OSM: Oncostatin M
PI3K: Phosphatidylinositol-3-kinase
Plin2: Perilipin 2
Pnlip: Pancreatic lipase
Ppargc1a: Peroxisome proliferator-activated receptor gamma coactivator 1 alpha
Ptpre: Protein tyrosine phosphatase receptor type E
qPCR: Quantitative polymerase chain reaction
RT: Reverse transcription
SOCS3: Suppressor of cytokine signaling 3
STAT3: Signal transducer and activator of transcription 3
STRING: Search Tool for the Retrieval of Interacting Genes
TLR: Toll like receptor
