# Correction to: Leukemia inhibitory factor inhibits erythropoietin-induced myelin gene expression in oligodendrocytes

**DOI:** 10.1186/s10020-020-00189-9

**Published:** 2020-06-25

**Authors:** Georgina Gyetvai, Cieron Roe, Lamia Heikal, Pietro Ghezzi, Manuela Mengozzi

**Affiliations:** 1 Department of Clinical and Experimental Medicine, Brighton & Sussex Medical School, Brighton, BN1 9PS UK; 2 Department of Pharmaceutics, Faculty of Pharmacy, Alexandria University, Alexandria, 21521 Egypt

**Correction to: Mol Med (2018) 24:51**

**https://doi.org/10.1186/s10020-018-0052-3**

Following publication of the original article (Gyetvai et al. 2018), the author Lamia Heikal would like to update her affiliation by adding a new one. The updated author affiliation is shown in this erratum.

Correct:

^1^Department of Clinical and Experimental Medicine, Brighton & Sussex Medical School, Brighton BN1 9PS, UK

^2^Department of Pharmaceutics, Faculty of Pharmacy, Alexandria University, Alexandria, 21521, Egypt
